# 428. Deep-Learning Based Predictive Model for Patients with Positive MRSA Cultures Using Time-Series Electronic Health Records

**DOI:** 10.1093/ofid/ofac492.503

**Published:** 2022-12-15

**Authors:** Masayuki Nigo, Laila Rasmy, Ziqian Xie, Edward J Septimus, Degui Zhi

**Affiliations:** UT Health Mc Govern Medical School, Houston, Texas; UTHealth School of Biomedical Informatics, Houston, Texas; UTHealth School of Biomedical Informatics, Houston, Texas; Texas A&M University System Health Science Center, Houston, Texas; UTHealth School of Biomedical Informatics, Houston, Texas

## Abstract

**Background:**

Methicillin-resistant *Staphylococcus aureus* (MRSA) is one of the common pathogens leading to significant morbidity and mortality in the hospital. This pathogen requires specific empirical antibiotics. Hence, identifying the personalized risks of this pathogen likely optimizes the usage of those antibiotics. Deep-learning based (DL) models are shown to be useful for modeling time-series electronic health record (EHR) data, and thus offering potential for predicting individualized risk for MRSA infection.

**Methods:**

Longitudinal data were retrospectively retrieved from EHR in Memorial Hermann System, Houston, Tx. Patient encounters, demographics, diagnostic & procedure codes, antibiotics use, and microbiology data were extracted between 1/2018 and 4/2021. Inpatient and outpatient data were included. We randomly identified roughly equal numbers of patients with positive culture (Cx) for MRSA, MSSA, other pathogens, and negative Cx. We set a 2-week window for the prediction, and any first culture within the window was used as an index Cx. Some patients had multiple predictions over time and were included into both MRSA vs. non-MRSA groups. Our team developed a DL model platform (Pyorch_EHR) for clinical outcomes predictions using structured EHR data. In this project, the outcome is MRSA positivity taken within 2 weeks from the index Cx. Datasets are split into 50, 30, and 20% for training, validation, and test, respectively. Other machine learning models; logistic regression (LR) and light GBM (LGBM) were used for comparison. Pytorch ver. 1.7.1 and Sklearn ver. 0.24.2 are used.

**Results:**

A total of 8164 patients and 22,563 patients were identified as MRSA and non-MRSA groups, respectively. Table 1 summarizes the key features of the patient’s characteristics. After model training using train and validation datasets, our model achieved an AUC of 91.8 in test dataset, whereas AUC of 86.0 and 88.2 in LR and LGBM, respectively. Figure 1 shows the cumulative incidence of 2 week MRSA positivity. Our model precisely stratified the risks.

**Figure:**
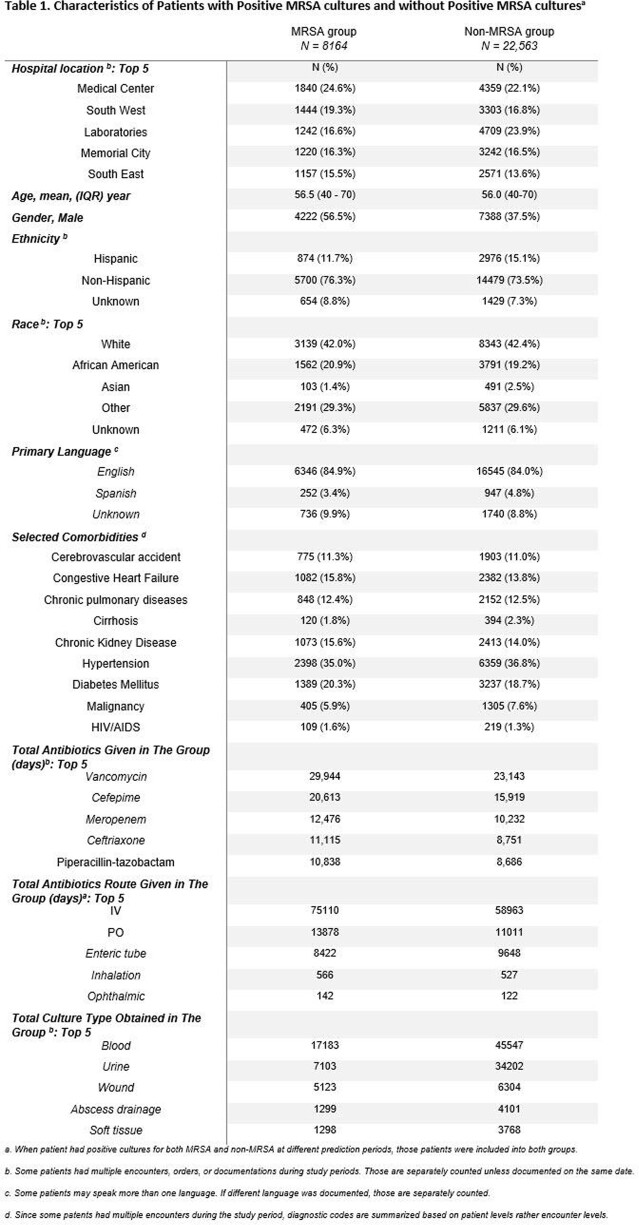

**Figure 1:**
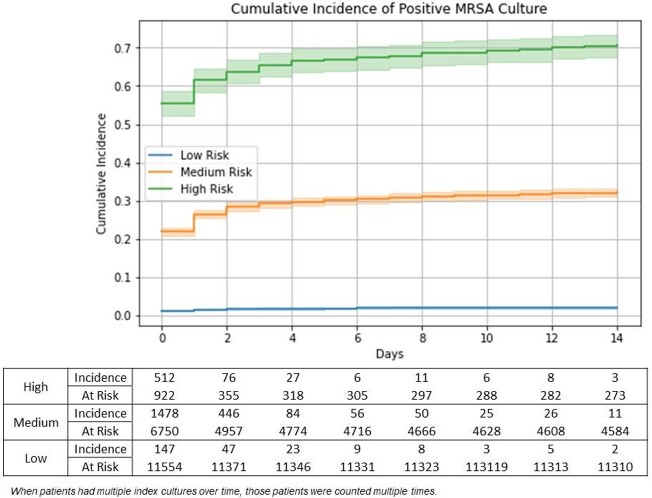
Cumulative Incidence of Positive MRSA Cultures Based on Risk Stratificationa

**Conclusion:**

Our DL based model exhibited excellent performance in the prediction of 2 week MRSA positivity. Our model precisely categorized patients into low, medium, and high-risk. This work should be validated with other data sources and high-risk subgroups.

**Disclosures:**

**All Authors**: No reported disclosures.

